# Efficacy of early antifibrotic treatment for idiopathic pulmonary fibrosis

**DOI:** 10.1186/s12890-021-01595-3

**Published:** 2021-07-10

**Authors:** Keishi Sugino, Hirotaka Ono, Natsumi Watanabe, Masahiro Ando, Eiyasu Tsuboi, Sakae Homma, Kazuma Kishi

**Affiliations:** 1 Department of Respiratory Medicine, Tsuboi Hospital, 1-10-13, Nagakubo, Asakamachi, Koriyama City, Fukushima 963-0197 Japan; 2 Department of Advanced and Integrated Interstitial Lung Diseases Research, School of Medicine, Toho University, 5-21-16, Omori-nishi, Ota-ku, Tokyo 143-8540 Japan; 3 Department of Respiratory Medicine, Toho University Omori Medical Center, 6-11-1, Omori-nishi, Ota-ku, Tokyo 143-8541 Japan

**Keywords:** Antifibrotic drug, Desaturation, GAP staging, Idiopathic pulmonary fibrosis, Prognosis

## Abstract

**Background:**

Although antifibrotic drugs, including nintedanib and pirfenidone, slow the progression of idiopathic pulmonary fibrosis (IPF), there is little data about the timing of start of antifibrotic treatment in real-world clinical practice. The present study aimed to clarify the efficacy of nintedanib and pirfenidone in patients with early-stage IPF.

**Methods:**

We compared survival and disease progression between patients with IPF with Japanese Respiratory Society (JRS) disease severity system stage I with and without oxygen desaturation on the 6-min walk test (6MWT) and increased the gender–age–physiology (GAP) staging. We examined the efficacy of antifibrotic drugs in patients with early-stage IPF.

**Results:**

The severity of stage I IPF (n = 179) according to the JRS criteria consisted of the following GAP staging criteria: stage I, 111 cases; stage II, 58 cases; stage III, 10 cases. The duration from the initial visit to disease progression and survival time was significantly shorter in JRS stage I patients with oxygen desaturation on the 6MWT or with increased GAP staging (unfavorable group) compared with patients without those factors. In the unfavorable group, the relative decline in percentage predicted forced vital capacity (%FVC) over 6 months was significantly lower in patients undergoing antifibrotic treatment compared with non-treated patients.

**Conclusion:**

Antifibrotic drugs have a beneficial effect on the decline in %FVC in Japanese patients with early-stage IPF who have oxygen desaturation on the 6MWT or increased GAP staging.

## Introduction

Idiopathic pulmonary fibrosis (IPF) is a fatal and progressive lung disease of undetermined etiology that has a heterogeneous clinical course. The median survival time from diagnosis is 3–5 years [1, 2]. Although there are no cures and few treatment options for patients with IPF, nintedanib and pirfenidone significantly reduce the decline in forced vital capacity (FVC) and have been approved for the treatment of IPF [3, 4]. Indeed, more recent IPF registries have demonstrated that the use of antifibrotic drugs (pirfenidone or nintedanib) are associated with improved survival of IPF patients in the era of antifibrotic therapies [5, 6]. Several retrospective and prospective studies have shown that a 5–10% decline in FVC within 6 or 12 months is associated with a significant increase in mortality [7–9]. Thus, we propose that antifibrotic treatment for IPF should be started as soon as the diagnosis is made. However, a “watch and wait” approach is not uncommon and the question of when to start antifibrotic treatment is still widely debated [10].

In Japan, IPF disease severity is assessed using the Japanese Respiratory Society (JRS) disease severity system, which is based on the partial pressure of oxygen in arterial blood (PaO_2_) at rest and the presence of oxygen desaturation during the 6-min walk test (6MWT) [11]. On the other hand, the gender–age–physiology (GAP) staging system is developed as a mortality risk prediction tool in IPF [12].

Recently, a post-hoc analysis of pooled date from the INPULSIS trials suggested that nintedanib had a similar beneficial effect on the rate of decline in FVC in patients with GAP stage I versus patients with GAP stage II/III at baseline [13].

To our best knowledge, little has been reported on the appropriate time to start antifibrotic treatment in Japanese patients with early-stage IPF. Herein, we aimed to clarify the efficacy of antifibrotic drugs, including nintedanib and pirfenidone, for patients with early-stage IPF in real-world clinical practice.

## Methods

### Study population

**Fig. 1:**
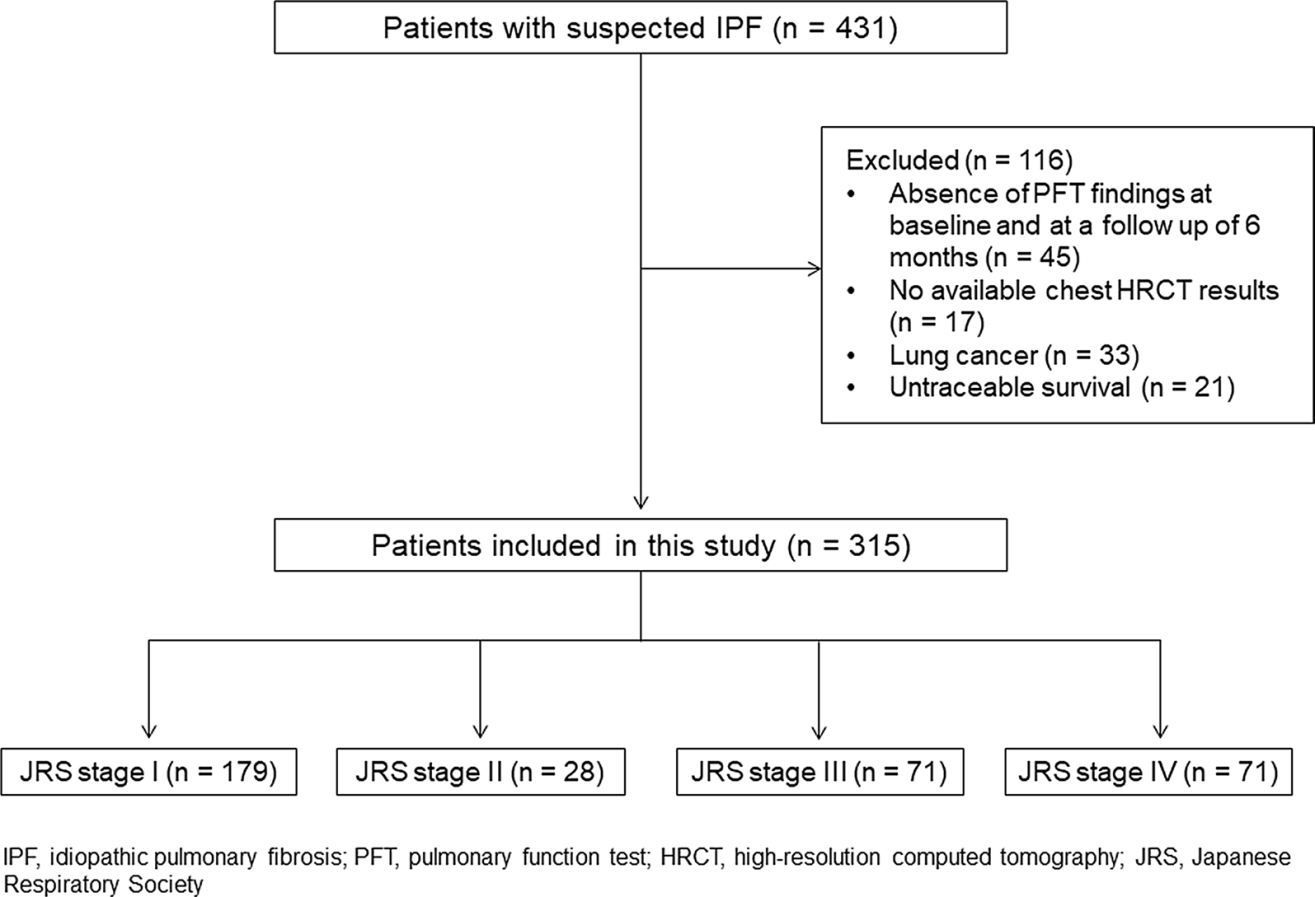
Flowchart showing inclusion and exclusion of patients

In this retrospective study, patients with IPF at two centers in Japan (Tsuboi Hospital and Toho University Omori Medical Center) were enrolled from April 2006 to March 2019. IPF was diagnosed based on 2011 international IPF guidelines [1]. The diagnosis of emphysema was based on upper lobe predominant and scattered distribution of low attenuation areas on chest HRCT, either no wall or with wall of less than 1 mm in thickness [14]. Combined pulmonary fibrosis and emphysema (CPFE) was defined as concomitant IPF and ≥ 10% emphysema on chest HRCT by criteria proposed by Ryerson, et al. [15]. Based on these findings, we excluded patients with CPFE in this study. The diagnosis of all patients was evaluated by a multidisciplinary team based on patients’ clinical, radiological and/or pathological findings. In total, 431 patients with suspected IPF were enrolled. Patients with absence of pulmonary function test findings at baseline and at a follow-up of 6 months (n = 45), unavailable chest high-resolution computed tomography (HRCT) results (n = 17), lung cancer (n = 33), and untraceable survival (n = 21) were excluded. A total of 315 patients were included. The number of patients with JRS stage I, II, III, and IV was 179, 28, 71, and 71, respectively (Fig. 1).

The modified diagnostic criteria for acute exacerbation (AE) of IPF proposed by Collard et al. [16] was as follows: (1) previous/concurrent IPF diagnosis; (2) unexplained worsening or development of dyspnea within 30 days; (3) chest HRCT with new bilateral ground-glass opacities and/or consolidation superimposed on a reticular or honeycomb background; (4) exclusion of alternative causes of acute lung injury such as pneumonia, left-sided heart failure, and pulmonary embolism.

Clinical and laboratory data was obtained from medical records, including IPF disease severity, efficacy of antifibrotic treatment, changes in pulmonary function (every 6 months), and prognosis. In Japan, classification of IPF disease severity in IPF is as follows: stage I (PaO_2_ ≥ 80 Torr at rest), stage II (80 > PaO_2_ ≥ 70 Torr at rest), stage III (70 > PaO_2_ ≥ 60 Torr at rest), and stage IV (PaO_2_ < 60 Torr at rest). If patients with stage II or III have oxygen desaturation (the lowest SpO_2_ is < 90%) during the 6MWT, they are classified as stage III or IV, respectively [11]. The GAP score and stage were calculated according to a method described by Ley et al. [12].

In this study, pirfenidone and nintedanib have been used since 2008, and 2015, respectively. In addition, patients who were diagnosed as IPF from 2006 to 2008, were treated as non-treated group. To evaluate the response to treatment, we defined disease progression as a relative decline of ≥ 5% and stable disease as a relative decline of < 5% in percentage predicted FVC (%FVC) over a period of 6 months.

All methods were carried out in accordance with relevant guidelines and regulations.

The study was approved by the Institutional Review Board of Tsuboi Hospital (no. Zizan 1-3). The Ethical Committees of Tsuboi Hospital waived the need for informed consent because of the retrospective clinical review.

### Chest computed tomography (CT) scan

Helical CT scanners (Aquilion 16, Toshiba, Tokyo, Japan and Aquilion Prime, Canon, Tokyo, Japan) were used. Thin-section CT images were obtained during full inspiration, and the scanning protocol consisted of reconstruction of a 1- to 2-mm slice thickness with a high spatial frequency algorithm. Thin-section chest CT images were photographed at window settings appropriate for the lung parenchyma (window level, − 600 to − 450 HU; width, 1600–1900 HU) in all patients.

### Pulmonary function test

Spirometry and measurements of diffusing capacity of the lungs for carbon monoxide (DLco) were performed using a pulmonary function test (PFT) system (Chestac-33, CHEST Co. Ltd., Tokyo, Japan). The diffusion capacity was measured using the single breath technique. These PFTs were performed by two technicians according to the method described in the American Thoracic Society criteria [17].

### 6MWT

The 6MWT was performed according to the ATS recommendations [17], and continuous monitoring of heart rate and oxygen arterial saturation with pulse oximetry, and walking distance were recorded. During 6MWT, patients were instructed to walk as fast as possible and were allowed to slow down or to stop if necessary. The oxygen desaturation on the 6MWT was defined as indicating < 90% on oxygen saturation with pulse oximetry during 6MWT.

### Statistical analysis

Data is presented as the number of patients with percentage or mean with standard deviation, as appropriate. The comparisons between the two groups were evaluated using Pearson’s Chi-squared test or Fisher’s exact test for categorical variables and the Student’s *t*-test for parametric continuous variables. Survival curves and the cumulative incidence of disease progression were estimated using the Kaplan–Meier method, and differences in the curves were calculated using the log-rank test. A two-sided *p* value of < 0.05 was considered statistically significant. Statistical analyses were performed using JMP statistical software (version 10.0.0, SAS Institute, Cary, NC, USA).

## Results

### Baseline demographic characteristics of patients with stage I IPF according to the JRS severity system

The study population consisted of 179 patients [148 males (83%) and 31 females (17%)] with a mean age of 71.5 years (range 43–86 years). One hundred fifty seven patients had a smoking history. Mean PaO_2_ was 90.3 Torr in room air. The percentage of patients with oxygen desaturation on the 6MWT was 37.4%. The mean %FVC and predicted DLco (%DLco) were 81.5% (range 37.4–129.1%) and 68.8% (range 23.7–143.2%), respectively (Table 1).

**Table 1:** Baseline demographic characteristics of patients with IPF with a disease severity of stage I according to the JRS severity system

Variable	Mean ± SD or number	Range
Age, years	71.5 ± 7.0	43–86
Sex, male/female	148/31	
BMI, kg/m^2^	21.6 ± 4.3	14.0–32.6
Smoking history, N/F/C	22/121/36	
GAP staging (I/II/III)	111/58/10	
mMRC (0/1/2/3/4)	28/72/62/13/3	
PaO_2_, Torr	90.3 ± 9.1	62.7–127
%FVC, %	81.5 ± 19.2	37.4–129.1
%DLco, %	68.8 ± 21.3	23.7–143.2
Desaturation on 6MWT, ±	73/106	
KL-6, U/ml	938 ± 586	193–3374
SP-D, ng/ml	271 ± 216	36.7–1390
Histological UIP diagnosis (%)	40 (22.3%)	

Data is presented as mean ± standard deviation. IPF, idiopathic pulmonary fibrosis; JRS, Japanese Respiratory Society; BMI, body mass index; N/F/C, never/former/current; GAP, gender–age–physiology; mMRC, modified Medical Research Council; PaO_2_, partial pressure of oxygen in arterial blood; FVC, forced vital capacity; DLco, diffusion capacity of the lungs for carbon monoxide; 6MWT, 6-min walk test; KL-6, Krebs von den Lungen-6; SP-D, surfactant protein-D; UIP, usual interstitial pneumonia

### Correlation between the numbers of patients with different JRS disease severities according to the JRS severity system and the GAP staging model

JRS stage I (n = 179) consisted of the following GAP staging model breakdown: stage I, 111 cases; stage II, 58 cases; stage III, 10 cases. The percentage of patients with GAP stage II/III with JRS stage I was 37.9% (Table 2).

**Table 2:** Correlation between the numbers of patients with different disease severities according to the JRS severity system and GAP staging model

	JRS severity system			
	Stage I	Stage II	Stage III	Stage IV
*GAP staging model*				
Stage I	111	14	34	7
Stage II	58	4	24	22
Stage III	10	0	13	18

JRS, Japanese Respiratory Society; GAP, gender–age–physiology

### Comparison of clinical and demographic characteristics between JRS stage I with and without oxygen desaturation on the 6MWT

Baseline GAP staging, modified Medical Research Council (mMRC) value, serum Krebs von den Lungen (KL)-6, and serum surfactant protein (SP)-D in patients with JRS stage I with oxygen desaturation were significantly higher compared with patients with JRS stage I without oxygen desaturation on the 6MWT, and PaO_2_, %FVC, %DLco, 6-min walking distance (6MWD), and lowest percutaneous oxygen saturation (SpO_2_) on the 6MWT were significantly lower for patients with JRS stage I with oxygen desaturation on the 6MWT compared with patients with JRS stage I without oxygen desaturation on the 6MWT (Table 3).

**Table 3:** Comparison of JRS stage I with and without oxygen desaturation on the 6MWT

	JRS stage I + desaturation (–)	JRS stage I + desaturation (+)	*P* value
Age, years	71.2 ± 6.9	71.9 ± 7.1	0.51
Sex, male/female	89/17	58/15	0.55
BMI, kg/m^2^	22.1 ± 3.8	21.3 ± 4.3	0.22
Smoking history, N/F/C	16/71/19	6/50/17	0.31
GAP staging (I/II/III)	80/24/2	31/34/8	< 0.0001
mMRC (0/1/2/3/4)	24/50/28/3/1	4/23/34/10/2	< 0.0001
PaO_2_, Torr	91.7 ± 9.1	88.0 ± 8.4	0.007
%FVC, %	86.9 ± 18.4	73.7 ± 17.7	< 0.0001
%DLco, %	76.2 ± 21.6	57.9 ± 15.4	< 0.0001
KL-6, U/ml	797 ± 533	1140 ± 603	< 0.0001
SP-D, ng/ml	221 ± 139	342 ± 278	0.0002
6MWD, m	400 ± 95	368 ± 106	0.03
Lowest SpO_2_ on 6MWT, %	93.1 ± 2.2	85.9 ± 3.7	< 0.0001

Data is presented as mean ± standard deviation. JRS, Japanese Respiratory Society; BMI, body mass index; N/F/C, never/former/current; GAP, gender–age–physiology; mMRC, modified Medical Research Council; PaO_2_, partial pressure of oxygen in arterial blood; FVC, forced vital capacity; DLco, diffusion capacity of the lungs for carbon monoxide; KL-6, Krebs von den Lungen-6; SP-D, surfactant protein-D; 6MWD, 6-min walking distance; SpO_2_, percutaneous oxygen saturation; 6MWT, 6-min walk test

**Fig. 2:**
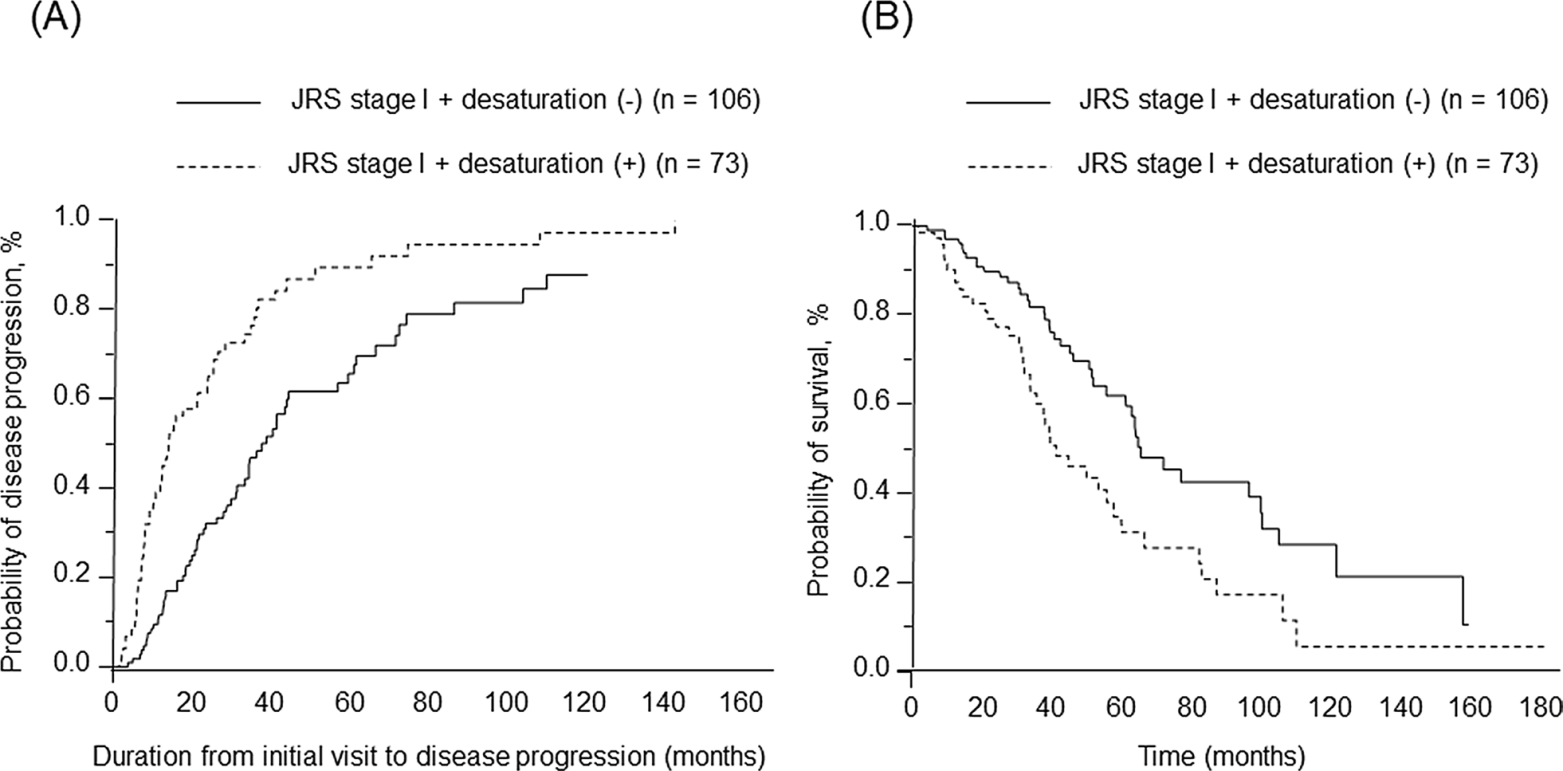
Comparison with disease progression and survival between patients with JRS stage I with and without oxygen desaturation on the 6MWT. (**A**) Kaplan Meier analysis for duration from initial visit to disease progression. (**B**) Kaplan Meier analysis for overall survival

The duration from the initial visit to disease progression was shorter in patients with JRS stage I with oxygen desaturation on the 6MWT compared with patients with JRS stage I without oxygen desaturation on the 6MWT [median survival time (MST), 13.9 months vs. 37.4 months, *P* = 0.002] (Fig. 2A). The Kaplan–Meier survival curve showed a significantly poorer outcome in patients with JRS stage I with oxygen desaturation on the 6MWT compared with patients with JRS stage I without oxygen desaturation on the 6MWT (MST = 40.8 months vs. 64.9 months, *P* = 0.002) (Fig. 2B).

### Comparison of clinical and demographic characteristics between patients with JRS stage I with GAP stage I and GAP stage II/III

The mean age, ratio of male, GAP staging, mMRC, and serum SP-D were significantly higher in patients with JRS stage I with GAP stage II/III compared with patients with JRS stage I with GAP stage I. In contrast, body mass index, %FVC, %DLco, 6MWD, and lowest SpO_2_ on the 6MWT were significantly lower in patients with JRS stage I with GAP stage II/III compared with patients with JRS stage I with GAP stage I (Table 4).

**Table 4:** Comparison of JRS stage I with GAP stage I and GAP stage II/III

	JRS stage I + GAP stage I	JRS stage I + GAP stage II/III	*P* value
Age, years	70.3 ± 7.1	73.3 ± 6.4	0.004
Sex, male/female	86/25	61/7	0.04
BMI, kg/m^2^	22.4 ± 3.8	20.8 ± 4.2	0.009
Smoking history, N/F/C	15/70/26	7/51/10	0.24
GAP staging (I/II/III)	111/0/0	0/58/10	< 0.0001
mMRC (0/1/2/3/4)	26/56/24/4/1	2/17/38/9/2	< 0.0001
PaO_2_, Torr	90.9 ± 9.3	89.1 ± 8.4	0.18
%FVC, %	91.3 ± 13.9	65.6 ± 15.9	< 0.0001
%DLco, %	76.1 ± 19.4	56.2 ± 18.5	< 0.0001
KL-6, U/ml	890 ± 546	1016 ± 643	0.16
SP-D, ng/ml	233 ± 134	333 ± 295	0.002
6MWD, m	417 ± 92	336 ± 94	< 0.0001
Lowest SpO_2_ on 6MWT, %	91.4 ± 3.9	88.2 ± 4.9	< 0.0001

Data is presented as mean ± standard deviation. JRS, Japanese Respiratory Society; BMI, body mass index; N/F/C, never/former/current; GAP, gender–age–physiology; mMRC, modified Medical Research Council; PaO_2_, partial pressure of oxygen in arterial blood; FVC, forced vital capacity; DLco, diffusion capacity of the lungs for carbon monoxide; KL-6, Krebs von den Lungen-6; SP-D, surfactant protein-D; 6MWD, 6-min walking distance; SpO_2_, percutaneous oxygen saturation; 6MWT, 6-min walk test

**Fig. 3:**
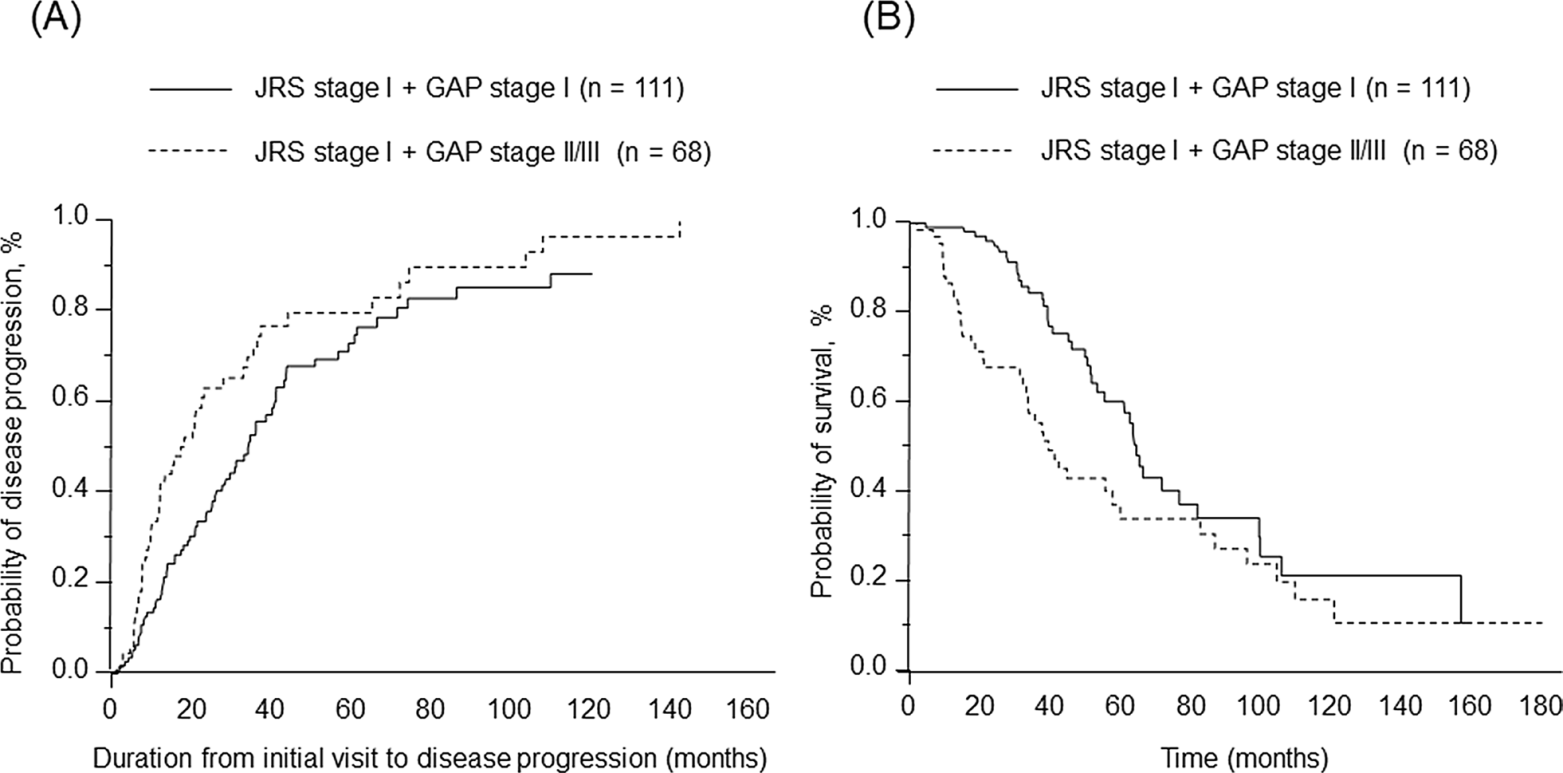
Comparison with disease progression and survival between patients with JRS stage I with GAP stage I and GAP stage II/III. (**A**) Kaplan Meier analysis for duration from initial visit to disease progression. (**B**) Kaplan Meier analysis for overall survival

The duration from the initial visit to disease progression was shorter in patients with JRS stage I with GAP stage II/III compared with patients with JRS stage I with GAP stage I (MST = 17.3 months vs. 34.4 months, *P* = 0.006) (Fig. 3A). The survival time for patients with JRS stage I with GAP stage II/III was significantly shorter compared with patients with JRS stage I with GAP stage I (MST = 39.1 months vs. 64.1 months, *P* = 0.004) (Fig. 3B).

### Antifibrotic treatment for patients with JRS stage I with oxygen desaturation on the 6MWT and JRS stage I with GAP stage II/III

Forty eight patients with JRS stage I with oxygen desaturation on the 6MWT and 39 patients with JRS stage I with GAP stage II/III were treated with antifibrotic drugs during 6 months. Fifteen of 48 patients with JRS stage I with oxygen desaturation on the 6MWT and 11 of 39 patients with JRS stage I with GAP stage II/III were treated with nintedanib, and each remaining patient received pirfenidone. There was no difference in comparative clinical and demographic characteristics between antifibrotic drug-treated patients and non-treated patients in JRS stage I with oxygen desaturation on the 6MWT and JRS stage I with GAP stage II/III (Tables 5, 6).

**Table 5:** Comparison of clinical and demographic characteristics between antifibrotic drug-treated group and non-treated group in JRS stage I with oxygen desaturation on the 6MWT

	Non-treated group	Treated group	*P* value
Age, years	71.4 ± 6.7	71.2 ± 6.9	0.91
Sex, male/female	28/9	40/8	0.42
Smoking history, N/F/C	4/26/7	5/31/12	0.44
GAP staging (I/II/III)	15/18/4	17/26/5	0.27
PaO_2_, Torr	89.1 ± 7.9	87.1 ± 7.0	0.22
%FVC, %	76.1 ± 19.7	70.0 ± 14.4	0.10
%DLco, %	58.7 ± 18.0	58.0 ± 18.3	0.85
KL-6, U/ml	1006 ± 543	1181 ± 678	0.20
SP-D, ng/ml	319 ± 247	331 ± 285	0.83
6MWD, m	365 ± 108	380 ± 102	0.50
Lowest SpO_2_ on 6MWT, %	86.9 ± 3.1	88.2 ± 5.0	0.16

Data is presented as mean ± standard deviation. JRS, Japanese Respiratory Society; N/F/C, never/former/current; GAP, gender–age–physiology; PaO_2_, partial pressure of oxygen in arterial blood; FVC, forced vital capacity; DLco, diffusion capacity of the lungs for carbon monoxide; KL-6, Krebs von den Lungen-6; SP-D, surfactant protein-D; 6MWD, 6-min walking distance; SpO_2_, percutaneous oxygen saturation; 6MWT, 6-min walk test

**Table 6:** Comparison of clinical and demographic characteristics between antifibrotic drug-treated group and non-treated group in JRS stage I with GAP stage II/III

	Non-treated group	Treated group	*P* value
Age, years	72.2 ± 5.4	73.2 ± 7.1	0.55
Sex, male/female	29/3	34/5	0.72
Smoking history, N/F/C	5/23/4	4/30/5	0.79
GAP staging (I/II/III)	0/27/5	0/32/7	0.62
PaO_2_, Torr	88.1 ± 9.8	87.9 ± 7.7	0.91
%FVC, %	68.2 ± 15.8	62.9 ± 13.5	0.13
%DLco, %	54.8 ± 21.2	55.0 ± 15.2	0.95
KL-6, U/ml	971 ± 660	1108 ± 699	0.40
SP-D, ng/ml	308 ± 291	348 ± 297	0.57
6MWD, m	351 ± 89	349 ± 101	0.92
Lowest SpO_2_ on 6MWT, %	89.4 ± 4.0	89.0 ± 5.4	0.76

Data is presented as mean ± standard deviation. JRS, Japanese Respiratory Society; N/F/C, never/former/current; GAP, gender–age–physiology; PaO_2_, partial pressure of oxygen in arterial blood; FVC, forced vital capacity; DLco, diffusion capacity of the lungs for carbon monoxide; KL-6, Krebs von den Lungen-6; SP-D, surfactant protein-D; 6MWD, 6-min walking distance; SpO_2_, percutaneous oxygen saturation; 6MWT, 6-min walk test

**Fig. 4:**
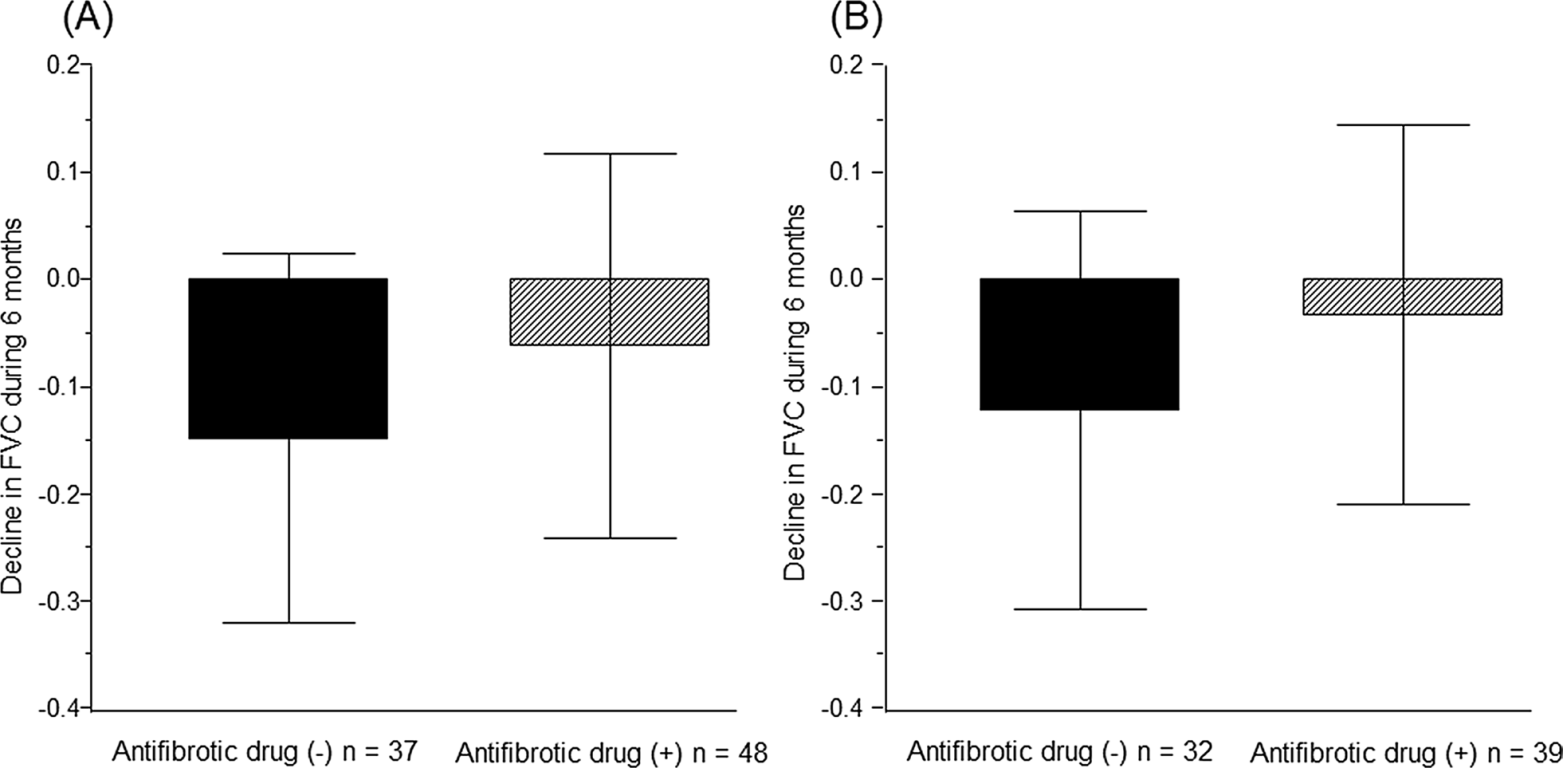
Comparison with the rate of decline in FVC over 6 months between the antifibrotic drug-treated group and the non-treated group in patients with JRS stage I with oxygen desaturation on the 6MWT or those with JRS stage I with GAP stage II/III. (**A**) In patients with JRS stage I with oxygen desaturation on the 6MWT. (**B**) In patients with JRS stage I with GAP stage II/III

**Fig. 5:**
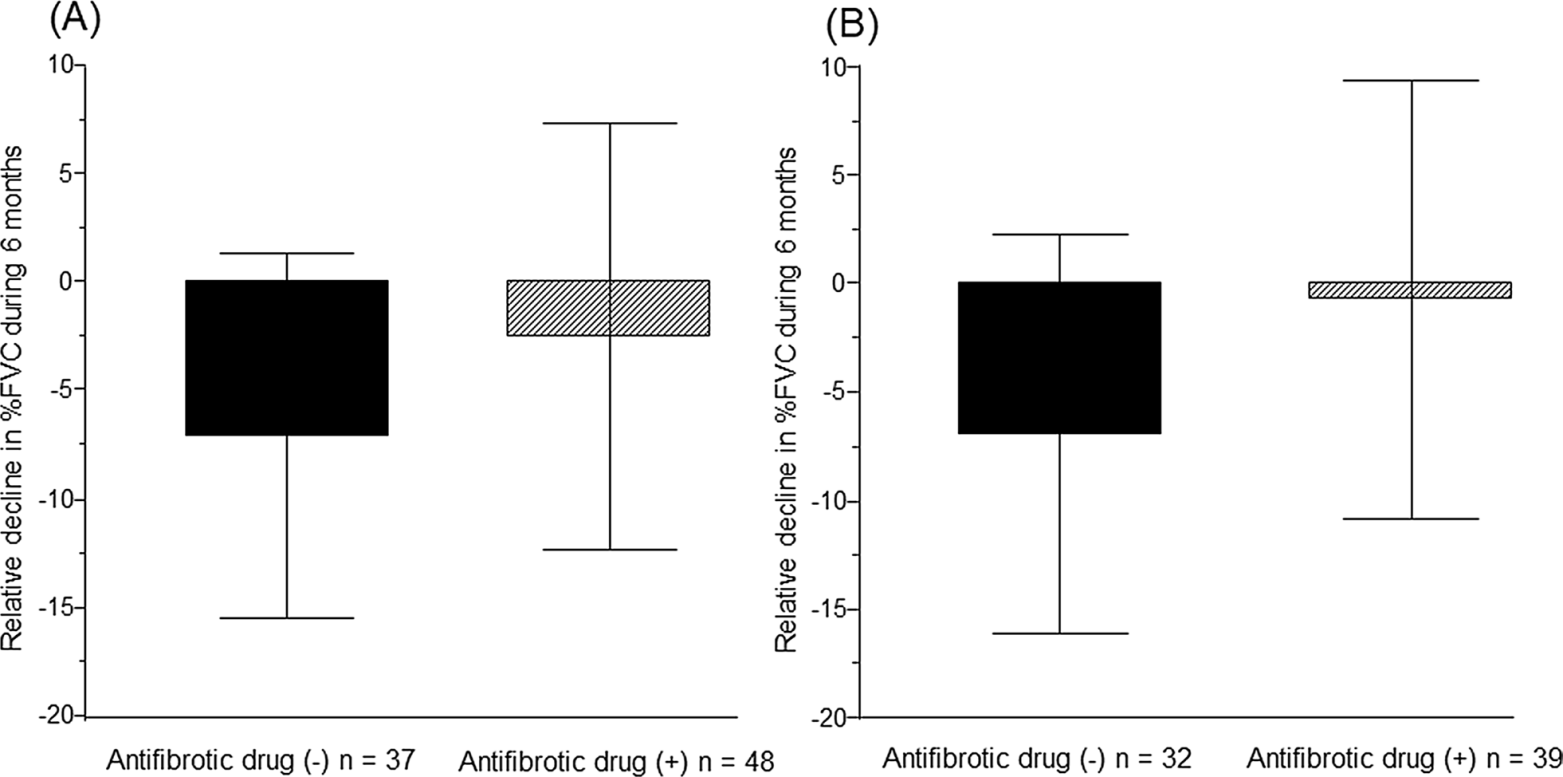
Comparison with the relative decline in %FVC over 6 between the antifibrotic drug-treated group and the non-treated group in patients with JRS stage I with oxygen desaturation on the 6MWT or those with JRS stage I with GAP stage II/III. (**A**) In patients with JRS stage I with oxygen desaturation on the 6MWT. (**B**) In patients with JRS stage I with GAP stage II/III

As a result, in patients with JRS stage I with oxygen desaturation on the 6MWT, the rate of decline in FVC over 6 months was − 61 mL ± 178 mL in the antifibrotic drug-treated group and − 147 mL ± 172 mL in the non-treated group (*P* = 0.02) (Fig. 4A). In patients with JRS stage I with GAP stage II/III, the rate of decline in FVC over 6 months was − 32 mL ± 176 mL in the antifibrotic drug-treated group and − 121 mL ± 185 mL in the non-treated group (*P* = 0.04) (Fig. 4B). Moreover, the relative decline in %FVC over 6 months was significantly lower in antifibrotic drug-treated patients compared with non-treated patients (JRS stage I with oxygen desaturation on the 6MWT: − 7.0% ± 8.4% vs − 2.4% ± 9.8%, *P* = 0.02; Fig. 5A, JRS stage I with GAP stage II/III: − 6.8% ± 9.2% vs − 0.6% ± 10.1%, *P* = 0.009; Fig. 5B).

Although there was no difference in comparative efficacy between antifibrotic drug-treated patients and non-treated patients (rate of disease progression in JRS stage I with oxygen desaturation on the 6MWT: antifibrotic drug-treated group vs. non-treated group, 41.6% vs. 62.1%, respectively, *P* = 0.08; JRS stage I with GAP stage II/III: antifibrotic drug-treated group vs. non-treated group, 38.4% vs. 53.1%, respectively, *P* = 0.23), the efficacy of antifibrotic treatments tended to be better.

## Discussion

Our data indicate that patients with IPF who have JRS stage I with oxygen desaturation on the 6MWT or increased GAP staging (II/III) had a significantly poorer prognosis compared with patients without these risk factors. Among these patients’ group, it is noteworthy that antifibrotic treatments had a beneficial effect on the rate of decline in FVC, as the relative decline in %FVC over 6 months was significantly lower in antifibrotic drug-treated patients compared with in non-treated patients. The findings support that treatment with antifibrotic drugs for IPF should be started in the early-stage patients with these conditions. However, delays in antifibrotic treatments are not uncommon, because some pulmonologists tend to fear adverse effects induced by antifibrotic drugs and thus postpone antifibrotic treatments until disease progression is observed [10, 18]. In particular, although medical cost subsidization for intractable diseases is essentially adapted to patients with stage III or IV disease in Japan, patients with IPF of JRS stage I or II are not covered immediately. Therefore, this limited subsidization system is one of the major causes of delay in the administration of antifibrotic treatments.

In Japan, the JRS severity system is used and is based on PaO_2_ and the presence of oxygen desaturation during the 6MWT to decide on medical cost subsidization for IPF. If patients with stage II or III disease present with oxygen desaturation during the 6MWT, they are classified into stage III or IV, respectively [11]. Kondoh et al. reported that no significant difference in survival between patients with JRS stage I and II IPF, although prognosis for patients with JRS stage IV was significantly poorer compared with patients with JRS stage III [19]. Thus, a revision of the JRS severity system was proposed. This revision stipulated that if patients with original JRS stage I demonstrate oxygen desaturation during the 6MWT, they are classified as JRS stage II. As a result, a significant difference in survival is observed between patients with revised JRS stage I and stage II. In the present study, patients with JRS stage I with oxygen desaturation on the 6MWT had a significantly worse prognosis compared with JRS stage I patients without oxygen desaturation.

The GAP staging model proposed by Ley et al. has been widely studied and validated in the United States, Italy, Korea, and Japan [12, 20, 21]. Recently, a Japanese study demonstrated the relationship of mortality risk with the JRS severity system and the GAP staging model [21]. According to the study, 64 patients (45.4%) with JRS stage I (n = 141) were classified into GAP stage II or III. In our study, overall survival in patients with JRS stage I IPF and GAP stage II or III [68 patients (37.9%) with JRS stage I (n = 179)] was significantly poorer compared with patients with JRS stage I and GAP stage I. Furthermore, they had a significantly shorter 6MWD, higher mMRC score, a lower SpO_2_, %FVC, and %DLco. Several baseline clinical characteristics including age, FVC, DLco, and oxygen desaturation on the 6MWT are reported to be prognostic factors for IPF [1]. These findings may indicate that damage to pulmonary function does not always reflect the PaO_2_ at rest. Therefore, the JRS staging system solely based on resting oxygenation may not be practically helpful. On the other hand, the GAP staging system is regarded as a well-validated staging system in IPF, because this system strongly correlates with disease severity and mortality. As a result, we believe that it is important to integrate oxygen desaturation on the 6MWT or the GAP staging with JRS stage I into the original JRS staging system.

Kolb et al. reported that treatment with nintedanib had the same annual rate of decline in FVC in patients with a predicted %FVC of > 90% and ≤ 90% [22]. Furthermore, Albera et al. described that no significant difference in the effect of treatment with pirfenidone between patients with more preserved (%FVC ≥ 80% or GAP stage I) versus less preserved lung function (%FVC < 80% or GAP stage II–III) [23]. Recently, a post-hoc analysis of pooled date from the INPULSIS trials suggested that nintedanib had a similar beneficial effect on the annual rate of decline in FVC in patients with GAP stage I versus GAP stage II/III at baseline [13]. These findings support the efficacy and safety of early antifibrotic treatments for IPF.

This study has some limitations. First, this was a retrospective study with a small sample size. Therefore, our results may not be representative of the entire IPF population in Japan. Second, although many studies have shown that a relative or absolute decline in predicted %FVC of ≥ 10% at 12 months is associated with a significant increase in mortality, we defined disease progression as a relative decline of ≥ 5% and stable disease as a relative decline of < 5% in predicted %FVC over a 6-month period. However, Zappala et al. described that a decline in predicted %FVC of 5–10% is related to an increase in the risk of mortality at 6 months [7]. Thus, we believe that a smaller decline in FVC may predict a worse prognosis more quickly. Third, we excluded patients who did not undergo PFTs at baseline and who did not undergo chest HRCT. Indeed, the considerable number of excluded patients may have led to selection bias.

In conclusion, our data suggests that antifibrotic drugs have a beneficial effect on the rate of decline in FVC in Japanese patients with early-stage IPF who demonstrated oxygen desaturation on the 6MWT or increased GAP staging. Further large prospective studies are needed in order to propose modified new staging system of IPF for future guidance of starting antifibrotic treatments.

## Data Availability

The datasets used and/or analyzed during the current study are available from the corresponding author on reasonable request.

## Abbreviations

IPF: Idiopathic pulmonary fibrosis
MST: Median survival time
HRCT: High-resolution computed tomography
AE: Acute exacerbation
BMI: Body mass index
mMRC: Modified Medical Research Council
KL-6: Krebs von den Lungen-6
SP-D: Surfactant protein-D
GAP: Gender–age–physiology
JRS: Japanese Respiratory Society
FVC: Forced vital capacity
%FVC: Percentage predicted FVC
DLco: Diffusing capacity of the lung for carbon monoxide
FEV_1_: Forced expiratory volume in 1 s
TLC: Total lung capacity
PFT: Pulmonary function test
PaO_2_: Partial pressure of oxygen in arterial blood
PaCO_2_: Partial pressure of carbon dioxide in arterial blood
CI: Confidence interval
SD: Standard deviation
